# Comparative Genomics and Genome Evolution

**DOI:** 10.2174/138920212799860715

**Published:** 2012-04

**Authors:** Sabyasachi Das, Masayuki Hirano

It has become a fundamental need to have genome sequence data due to the fact that the basic process of evolution is the change in DNA sequence and genome size. Recent advances in DNA sequencing technology have facilitated the availability of quite a large number of complete genome sequences from simplest prokaryotes to higher eukaryotes. The availability of whole genome sequence data at our fingertips can provide critical insight into how various genomic compositions have contributed to a contemporary understanding of molecular evolution. For this reason, comparative evolutionary genomics has become one of the most rapidly advancing disciplines in the biological sciences. The new generation of comparative genomics offers a powerful aid to studying evolutionary changes among organisms and identifying the genes that are conserved among species, and also the genes that give each organism its own specific characteristics. Informatics related to structural and functional genomics is potentially important in understanding the emergence of new phenotypic characters necessary for the adaptation of organisms. In addition, the evolutionary perspective of genes and genomes is also helpful in understanding disease susceptibility.

A large number of scientists from all over the world are involved in determining the genomic underpinnings of morphological, physiological, and behavioral changes. A lot of research has been done to find selection signals on a genome-wide scale, and also focusing on specific gene families to learn more about evolutionary mechanisms. Therefore, it is necessary to compile various current aspects of genomic and evolutionary research at some point. This prompts us to devote a special issue we have named “Comparative Genomics and Genome Evolution”.

The series of review articles in this issue of *Current Genomics* present current advances in multiple areas of comparative genomics and molecular evolutionary studies. This special issue is comprised of eight high quality articles which have been selected through a rigorous reviewing process. Several years ago, with the ground-breaking work of Max D. Cooper and colleagues, an adaptive immune system was discovered in a representative jawless vertebrate (lamprey). The first review article describes concordance and divergence in the immunogenetic architecture between two alternative adaptive immune systems of jawed and jawless vertebrates, and discusses in detail the evolution of the jawless vertebrate immune system on the basis of currently available lamprey genomic resources. In the second review paper, Nikolas Nikolaidis and colleagues discuss the current knowledge on the genomics and evolution of the immunoglobulin mulitigene family in vertebrates. To provide further coverage and discussion of the evolutionary dynamics of multigene families, we commissioned Yoshihito Niimura to review the evolutionary genomics of the olfactory receptor multigene family on a genome-wide scale. The fourth review article presents the interesting scenario of the evolution of mammalian sex chromosomes. Yoko Satta and colleagues reviewed evolutionary aspects related to genomic rearrangements and structures of the sex chromosomes. The next review article, by Arnab Gupta and Svetlana Lutsenko, focuses on the origin and evolution of copper transporting ATPases in eukaryotic organisms. In the sixth article, Mariko Kondo and Koji Akasaka highlight the phylogenetic relationships among echinoderms and the current status of echinoderm genome analysis. The seventh article is related to the evolution of microRNAs. In this article, Zhumur Ghosh and Bibekanand Mallick describe advances of genomics, evolution, and biogenesis of microRNAs. Finally, we conclude with an interesting review article by Chitra Dutta and Sandip Paul on microbial genome signature related to different lifestyles. The authors describe how closely related microbial species are diverged on the basis of the genomic signature of ecological kinship throughout microbial evolution.

Recognizing the growing interest in evolutionary genomics, we are happy to present this collection of high quality review articles for this special issue. We would like to thank all of the reviewers for their valuable comments meant to improve the quality of articles. In addition, we offer special thanks to the Editor-in-Chief, and the *Current Genomics *editorial/production staff, who have contributed invaluably to this project. We hope you find the issue timely, scholarly, and interesting.

